# Influence of flow rate and different size of suction cannulas on splatter contamination in dentistry: results of an exploratory study with a high-volume evacuation system

**DOI:** 10.1007/s00784-022-04525-7

**Published:** 2022-05-10

**Authors:** Christian Graetz, Viktor Hülsbeck, Paulina Düffert, Susanne Schorr, Martin Straßburger, Antje Geiken, Christof E. Dörfer, Miriam Cyris

**Affiliations:** Clinic of Conservative Dentistry and Periodontology, University Hospital of Schleswig-Holstein, Campus Kiel, Arnold-Heller-Str. 3 (Haus B), 24105 Kiel, Germany

**Keywords:** Experimental setup, Splatter contamination; SARS-CoV2, Tooth preparation, Air-polishing, Suction system

## Abstract

**Objectives:**

SOPs recommend high-volume evacuation (HVE) for aerosol-generating procedures (AGPs) in dentistry. Therefore, in the exploratory study, the area of splatter contamination (SCON in %) generated by high-speed tooth preparation (HSP) and air-polishing (APD) was measured when different suction cannulas of 6 mm diameter (saliva ejector (SAE)), 11 mm (HC11), or 16 mm (HC16) were utilized versus no-suction (NS).

**Materials and methods:**

Eighty tests were performed in a closed darkened room to measure SCON (1m circular around the manikin head (3.14 m^2^) via plan metrically assessment through fluorescence technique. HSP (handpiece, turbine (Kavo, Germany)) or APD (LM-ProPower^TM^ (Finland), Airflow®-Prophylaxis-Master (Switzerland)) for 6 min plus 5 s post-treatment were performed either without suction or with low-flow (150 l/min for SAE) or high-flow rate (250 l/min/350 l/min for HC11/HC16) suction. All tests were two-tailed (*p*≤0.05, Bonferroni corrected for multi-testing).

**Results:**

Irrespective the AGP, SCON was higher for NS (median [25th; 75th percentiles]: 3.4% [2.6; 5.4]) versus high-flow suction (1.9% [1.5; 2.5]) (*p*=0.002). Low-flow suction (3.5% [2.6; 4.3]) versus NS resulted in slightly lower but not statistically significantly lower SCON (*p*=1.000) and was less effective than high-flow suction (*p*=0.003). Lowest contamination values were found with HC16 (1.9% [1.5; 2.5]; *p*≤0.002), whereat no significant differences were found for HC11 (2.4% [1.7; 3.1]) compared to SAE (*p*=0.385) or NS (*p*=0.316).

**Conclusions:**

Within study’s limitations, the lowest splatter contamination values resulted when HC16 were utilized by a high-flow rate of ≥250 l/min.

**Clinical relevance:**

It is strongly recommended to utilize an HVE with suction cannulas of 16mm diameter for a high-flow rate during all AGPs and afterwards also to disinfect all surface of patients or operators contacted.

## Introduction

Independent various definitions exist for the terms “aerosol” and “splatter”; both were always indicated as possible risk for infections for dental staff [1] as they could contaminate with saliva and/or blood. Hence, it is not surprising that during the SARS-CoV-2 pandemic, dentistry was officially classified as one of the very high-risk occupations for transmission of the disease [2]. But is that assessment correct or too random in comparison to other medical specialties, especially as a paucity of robust data supporting some of these restrictions [3]? Yet, in dental practice, various fluid-cooled instruments were identified as aerosol-generating procedures (AGPs) and pose a potential risk to the patients and dental personnel; however, the exact infection dose required in virus copies to trigger an infection, e.g., with SARS-CoV-2, is currently unknown. Thus, adequate protective measures against pathogens transmitted via droplets, splatter, or aerosols from the patients’ oral cavity are always recommended in dentistry [4]. Especially during AGPs, it has to be considered that there is neither a great distance between the patient and the dentist’s face nor that patients get to wear masks during treatment. AGPs generate droplets with particle sizes of 0.5–20μm [5, 6], with the majority of the rebounding dental spray mist consisting of droplets larger than 10μm. Nearly 90% of these droplets settle as splatter on the patient’s face or body surface no later than some minutes after creation [2, 7, 8]. However, depending on the relative air humidity, larger droplets may transform into aerosol particles [9]. Without room air exchange, the average size of the droplets can be reduced from 12–21μm to about 4μm within 10 min [10]. This will be associated with a higher risk of infection [11]. A recently published experimental study from Vernon et al. [3] reported the aerosolization of active virus in a dental clinic as a marker for risk determination and found for their worst-case scenario for possible SARS-CoV-2 dispersal that the use of a high-speed contra-angle handpiece instead of a dental turbine for high-speed tooth preparation (HSP) in combination with a rubber dam or high-volume evacuation (HVE) can vastly reduce the risk of viral aerosolization in nearly 100%. Thus, a germane guideline recommended the risk-adapted combination (e.g., according to the current incidence of the region, the type and duration of required AGP) of different measures to minimize the risk of infection by airborne particles of all sizes, including intraoral suction with HVE in combination with suction cannula of diameter ≥10mm whenever possible [2]. However, the authors are aware that not always high-flow rates of the HVE and/or increased diameters of suction cannulas were routinely used.

Therefore, we aimed to identify the amount of splatter contamination (SCON in percent of a predefined area around the manikin head in real time) generated by two different AGPs under simulated standardized conditions: (1) HSP versus (2) air-polishing when an HVE with different types of cannulas was utilized versus no intraoral suction (control).

## Material and methods

### Experimental setup—manikin head and test dental procedure

A setup was chosen to measure the generated splatter and droplets larger than 100μm by different AGPs around a manikin head (Kavo, Biberach, Germany). Always, two investigators were inside the test room, one performed all dental procedures and the other one operated the measurement technology. At all times, every investigator wore a surgical mask (3M Deutschland GmbH, Neuss, Germany), whereat the operator wore additionally a face shield (Dental Design oHG, Bad Bramstedt, Germany) over the surgical mask according to internal guidelines for treating non-infection patients during AGPs.

### Aerosol-generating procedures

In total, eighty tests were performed. Each test of all AGPs took 6 min plus 5 s of post-treatment without AGP (in total, 365 s). During this observation time, six teeth had to be treated in the upper (tooth 16, 11, 24) and lower jaw (tooth 36, 41, 44). All tests were either done at 12 o’clock position or 8 o’clock position of the operator. The frequency of instrument, operators’ position, and utilized cannula/flow rate of the HVE device were randomized (Microsoft Excel 16, Microsoft Corporation, One Microsoft Way Redmond, WA, USA) for each of the two operators (C.G., M.C.) to avoid influence of training effects.

**Fig. 1:**
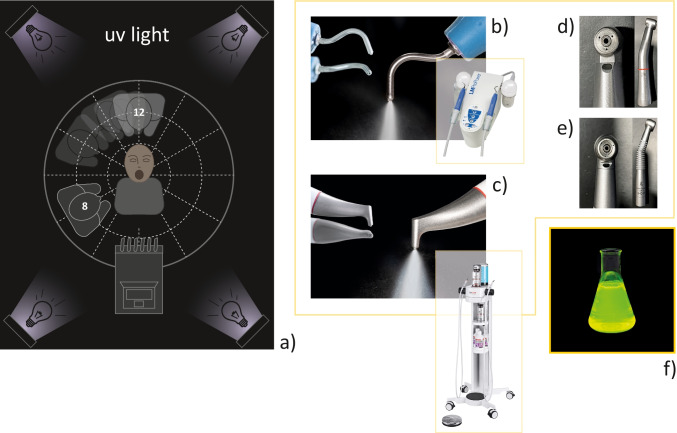
Schematic view of the (**a**) experimental setup from the camera position in a darkened room with the central manikin head, dental unit at 6 o’clock position, and the ultraviolet lights in four corners. On the right handside, the treatment devices used are shown: (**b**) LM-ProPower CombiLED (LM-Instruments, Oy, Pargas, Finland), (**c**) Airflow Prophylaxis Master (EMS, Nyon, Switzerland), the (**d**) high-speed contra-angle handpiece (Kavo, Biberach, Germany), and (**e**) dental turbine (Kavo, Biberach, Germany). The cooling water for all treatment devices was enriched with (**f**) 0.5g/l fluorescein (Uranin, Niepötter Labortechnik, Bürstadt, Germany)

Different treatment devices were applied for AGPs. HSP was performed either with a high-speed contra-angle handpiece with 250,000 rpm (Kavo, Biberach, Germany) or with a dental turbine (Kavo, Biberach, Germany) with 350,000 rpm. Both devices showed three water coolant ports (Fig. 1), and before each test, always the coolant flow of both HSP devices was measured.

Two air-polishing devices (APD) with non-abrasive powder (LM-Instruments Oy, Pargas, Finland; Airflow Prophylaxis Master, EMS, Nyon, Switzerland) were utilized on the middle level with handpieces for supragingival application (LM-Supra A; Airflow handpiece). The devices operated either with a glycine powder (particle size 25μm; LM-Glycin, LM-Instruments Oy, Pargas, Finland) or with erythritol powder (particle size 14μm; AIRFLOW® PLUS; EMS, Nyon, Switzerland). The powder-water flow was measured before each test.

All four instruments were used in line with the manufacturer’s specifications for tooth preparation or air-polishing.

### High-volume evacuation systems and suction cannula

Except for the negative control (no intraoral suction during AGP), for all other tests, a mobile dental unit (Galit Gallant Cart-5 Autonome, Ternopil, Ukraine) with an integrated HVE system (Dürr Dental SE, Bietigheim-Bissingen, Germany) for reproducible condition was utilized. Before every test, the flow rate of the HVE had been calibrated according to either 350 l/min, 250 l/min, or 150 l/min (measuring point: end of the suction tube at the base of the cannula/end of the tube).

**Fig. 2:**
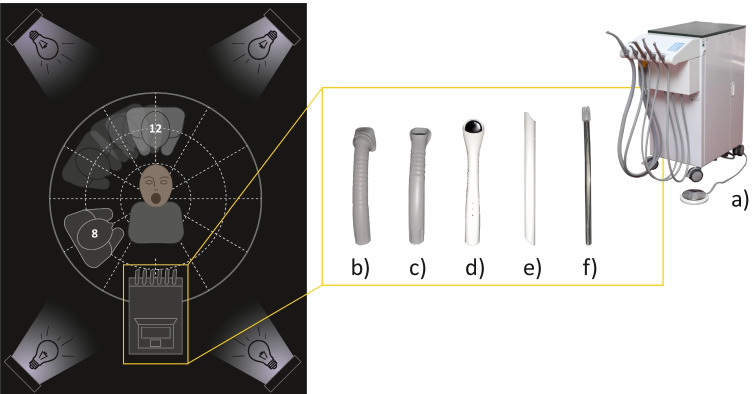
The (**a**) Dental unit (Galit Gallant Cart-5 Autonome Dental Unit, Ternopil, Ukraine) and the different suction cannulas were used: (**b**) 16-mm Prophylaxis cannula (Dürr, Bietigheim-Bissingen, Germany), (**c)** 16-mm Universal cannula Protect (Dürr, Bietigheim-Bissingen, Germany), (**d**) 16-mm Purevac HVE cannula (Dentsply/Sirona, Bensheim, Germany), (**e**) 11-mm Hygovac cannula (J.H. Orsing AB, Helsingborg, Sweden), and (**f**) 6-mm saliva ejector (Euronda, Vicenza, Italy)

As illustrated in detail in Fig. 2, five different intraoral suction cannulas were utilized: a 6-mm saliva ejector (SAE), a 11-mm suction cannula (HC11), and three types of 16-mm suction cannulas (HC16). Each cannula type was used with different flow rates: 150l/min for SAE only, while HC11 and HC16 were used with 250 l/min and 350 l/min, respectively.

### Treatment room and visualization of splatter contamination

**Fig. 3:**
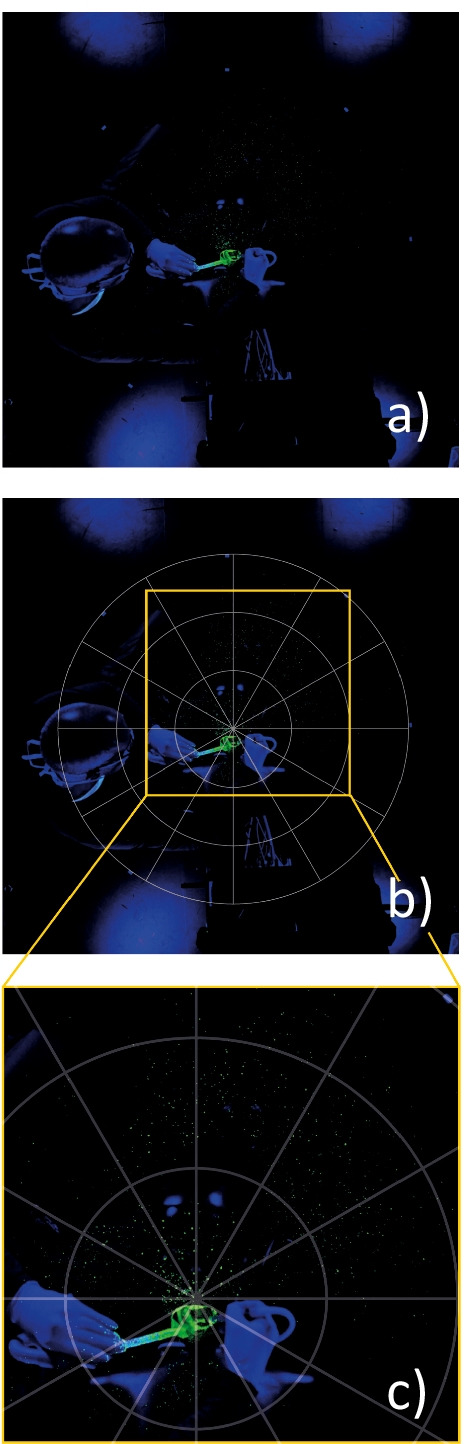
The plan metrically evaluation of splatter contamination on the basis of original photos and the respective schematic representation from the darkened room in camera view with different sections. **a** Application of an ADP device and the HC11 cannula utilized with the HVE from the operator 8 o’clock position in the original and as schematic representation. **b** Central area of interest with a radius of 1m aligned to the mouth of the manikin head and (**c**) a close-up of the original image (the fluorescent spots in the periphery are now clearly visible)

For the current investigation, a closed room (floor surface 12.1 m^2^) in the Clinic of Conservative Dentistry and Periodontology, University Medical Center Schleswig-Holstein, Kiel, Germany, was darkened and all surfaces/walls of the room, all dental devices, and the manikin head were wrapped with matt black foil (3M Deutschland GmbH, Neuss, Germany) or colored with black matt lacquer (Plasti Dip Deutschland GmbH, Aschaffenburg, Germany). To visualize the splatter contamination during treatment, 0.5 g/l fluorescein (Uranin, Niepötter Labortechnik, Bürstadt, Germany) was added to the water supply of all devices for AGP, which would fluoresce with bright yellow/green color when exposed to ultraviolet light (HY-FX80W-UV-B with 400–410nm, Shenzhen, China). Four lights were positioned in each corner of the room on the ground level of the manikin head, which allowed a complete illumination of the measurement area (area of interest, 3.14 m^2^ around the manikin head) in spite of the mobile examiner (Fig. 1). Therefore, not only deposited fluorescing material on all black surfaces, but also non-deposited airborne particles floating between the floor and the camera were visible in the photographs (Fig. 3).

All tests were performed without natural ventilation or air conditions at a constant temperature (mean (SD) 23.4 (0.3)°C) and with an air humidity of approximately 44.7 (1.1)% (TopMessage System, Delphin Technology, Bergisch Gladbach, Germany).

### Plan metric evaluation of splatter contamination

The evaluation of the contaminated area with splatter was plan metrically assessed. To enable standardized evaluation through the camera, the manikin head was placed over fixed marks on the ground in the treatment room for a reproducible position (Figs. 2 and 3). Due to the downward-facing camera (Canon, EOS D30, Tokyo, Japan) position in 2.34-m height, the surface directly underneath the manikin head was not measurable (Fig. 2). The camera was used with a 17–40-mm zoom (EFS, Canon, Tokyo, Japan); photographs of the area of interest (*n*=5920) were recorded (per trial: baseline and every 5s during 6min of AGP plus 5s post-treatment). Focus and position of the camera were checked on an extern monitor with the help of an open source software (qDslrDashboard V3.6.4. for macOS, https://dslrdashboard.info). After each test, the photographs were transferred to the evaluation program by means of digitizer software (Image J, NIH, Bethesda, USA). Based on these images, an evaluation of the splatter and droplets with a size ≥100μm (limited by the camera resolution) was then performed using digital image subtraction (Image J, NIH, Bethesda, USA) to calculate the splatter contaminated area (SCON in %) including the number and size of droplets. At least, the data of the calculated individual splatter distribution at every point of time during AGP were transferred to another program (SPSS Statistics 27, IBM, NY, USA) for statistical calculation.

### Outcomes and statistical analysis

As a primary outcome, SCON in a circle of 3.14m^2^ around the manikin head was determined and calculated as ΔSCON per 5s. The data analysts (V.H., C.G.) were blinded to the instruments, cannula, and flow rate used for the plan metric evaluation.

The number of samples (*n*=5920 photographs) was calculated according to a previously published investigation by our group [8]. However, we did not perform any power calculation before the study, and therefore, we aimed for a maximal statistical power no intragroup analyzes of high-flow suction, HSP, or ADP. Data acquisition, collection, and statistical analysis were done with SPSS Statistics (SPSS Statistics 27, IBM, Chicago, IL, USA). Normal distribution was tested by Kolmogorov-Smirnov and Shapiro-Wilk. There was no normal distribution. Subsequently, a mean value comparison was performed using the Kruskal-Wallis-test to detect significant differences according to SCON values among the three categories of suction cannula SAE, HC11, and HC16 and the control without HVE. The difference between HSP versus APD and among the type of suction cannulas, the Mann-Whitney-U test was used for subgroup analysis. All tests were two-sided; statistical significance was assumed if *p*≤0.05 (Bonferroni corrected for multi-test).

## Results

For both HSP devices, we measured nearly the double volume of coolant fluid (mean (SD) 73.1 (11.5) ml/min) compared to ADP with 34.3 (9.1) ml/min of water-powder fluid.

**Fig. 4:**
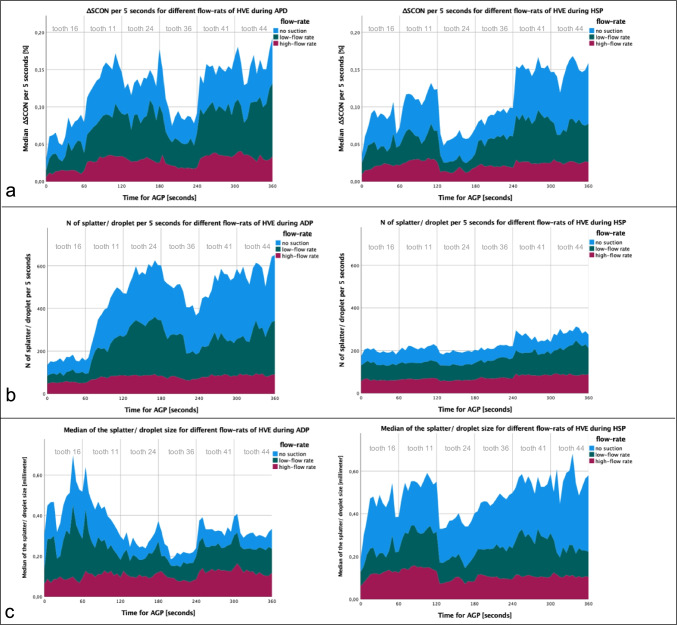
**a** The change splatter contamination area (ΔSCON) per 5s, (**b**) number, and (**c**) size of splatter/droplets measured as fluorescing particles per 5s for 6min of air-polishing (APD) versus high-speed tooth preparation (HSP) divided for no suction, low-flow suction (150l/min) with saliva ejector (SAE), or high-flow suction (≥250l/min) with suction cannulas of 11mm (HC11) or 16mm diameter (HC16) utilized by a high-volume evacuation system (HVE)

According to the descriptive evaluation of ΔSCON per 5 s, we found the highest result contamination when treating without suction the first right incisor in both jaws (upper/lower jaw, 60–120/240–300 s) independent of the AGP group (Fig. 4a). Lower ΔSCON values for both AGP groups were measured for the first molars (upper/lower jaw 0–60/180–240 s). The diagrams of figure 4a show further on that with higher flow rates of the HVE ΔSCON values per 5s will be decreased. In the category of high-flow rate, we found similar low contamination levels for ADP and HSP, whereat higher contamination resulted for ADP versus HSP in the categories of low-flow rate and no-suction (Fig. 4a). Similar results were measurable for the N of particles per 5 s, with nearly double the number of droplets and splatter induced by ADP versus HSP (Fig. 4b). However, the lowest number of particles resulted when high-flow rate suction with HVE was performed and the results for ADP and HSP were now nearly similar, again. Also, the lowest size with around 100–180μm of the induced splatter and droplets was found during high-flow rate suction for both AGP groups and increased up to 600μm during control tests without any intraoral suction (Fig. 4c). Surprisingly, for ADP, a decrease of droplet/splatter size during observation time was measurable in the categories of low-flow rate and no-suction.

In total, we found SCON was significantly higher for no-suction (median [25th; 75th percentiles] 3.4% [2.6; 5.4]) versus high-flow suction 1.9% [1.5; 2.5] (*p*=0.002) as well as low-flow suction with 3.5% (2.6; 4.3) less effective versus high-flow suction (*p*=0.003). In subgroup analysis, no significant differences were found between both AGP-groups for no-suction (APD/HSP 3.4% [2.8; 5.0]/3.8% [2.1; 5.5]; *p*=0.886), low-flow rate with SAE (APD/HSP 4.0% [3.2; 4.8]/3.0% [2.0; 3.8]; *p*=0.200) or high-flow rate of HC11 and HC16 with the HVE (APD/HSP 1.9% [1.6; 2.5]/1.8% [1.4; 2.5]; *p*=0.330). Irrespective of the AGP and different flow rates for HVE, the lowest contamination value results utilizing HC16 cannulas (1.9% [1.5; 2.5]; *p*<0.001). No significant differences were found for HC11 (2.4% [1.7; 3.1]) compared to control (*p*=0.316) or SAE (*p*=0.385). For details, see Table 1.

At least, we analyzed in a descriptive manner the spreading and direction of the AGP-generated splatter (no-suction tests). We found for ADP splatters/droplets in all four quarters around the manikin head versus HSP, with contamination only in the inner circle nearby the manikin head. HSP resulted in more than 60% of all sections with low particle contamination (*n*≤10 droplets/splatter per area).

## Discussion

As confirmed by several investigations [8, 12–14], we found that intraoral suction with cannulas of 16mm diameter and high-flow rate with ≥250l/min leads to lowest values of SCON for different AGPs. Also, we could show that a smaller line diameter and a saliva ejector lead to measurable flow rate limited by 150l/min in our study. This is not surprising, as without correction of the HVE’s suction pressure power, a smaller diameter will reduce the flow rate in total in a physical manner, and so, significantly higher SCON values will be occurring [8, 15]. There is no dispute that a saliva ejector could aspirate saliva and coolant fluid in the mouth, but them failed to eliminate spray mist and should use in addition to a suction cannula for high-flow rate suction. But oftentimes, HVE are optimized for vacuum and not for flow rate [15] or were limited for higher power as it will cause more noise [16]. But the flow rate at the opening end of each suction cannula is the crucial physical parameter for reducing spray mist [15]. In detail, the intraoral suction generates a counterflow, which in turn slows down the emitted splatter generated by AGP and has to be so strong that no spray mist leaves the mouth opening. Only a few studies could show such 100% mitigation of spray mist [3, 15], whereat the majority of studies including the current investigation failed to do so and recommended further interventions to control contamination [8, 14, 17, 18]. This includes among others rubber dam application, pre-procedural antimicrobial oral rinses, and an HVE as tested in our study [2, 19]. Hence, the HVE has the potential to minimize bio-aerosols generated by HSP or ADP, but it has to be right instructed to utilize it properly for high effective control of spray mist. For instance, optimal intraoral positioned suction cannulas significantly influence the results of spray mist reduction [8, 20].

Besides the statistically significant differences for effective mitigate spray mist due to flow rates and suction cannula is in line with the evidence according suction equipment in dentistry [16], we measured no difference between HSP versus ADP in total (Table 1). This is contrary to data from other investigation, which show higher prevalence of spray mist/splatter contamination for HSP with dental turbine versus high-speed contra-angle handpiece [3, 20] or for periodontal treatments with ADP versus ultrasonic scaling [17]. The systematic review of Innes et al. [21] described a hierarchy of AGP contamination risk: higher (ADP, HSP, ultrasonic scaling, air-water syringe, extractions using motorized handpieces); moderate (slow-speed handpieces, prophylaxis, extractions); and lower (air-water syringe [water only] and hand scaling). We have to assume that all performed AGPs produce enough (aerosol) particles/splatter to be a potential source of infection through inhalation or contact transmission [22]. Therefore, utilizing proper operating techniques, e.g., for ADP, while ensuring a maximum protection from aerosols for the patient and the operator was recommended for a long time [23]. On the other side, it should not be unmentioned that there are also efforts to reduce the spray mist during AGPs in total, e.g., by using more viscous coolant fluids, and that this could significantly reduce the amount of generated aerosols and splatter contamination distance [24]. Nevertheless, Farah et al. [24] gave no information how this will influence the change of the pulp temperature or the surrounding periodontal tissue.

**Table 1:** Comparison of groups of flow rate with different suction cannulas divided for high-speed tooth preparation (HSP) versus air-polishing (APD)

Groups of flow rate					
		SCON in % (median [25th; 75th percentiles]):	*p* value between no-suction and low-flow suction	*p* value between no-suction and high-flow suction	*p* value between low-flow-suction and high-flow suction
All AGP devices	No-suction	3.4 (2.6;5.4)	*p*=1.000*	*p*=0.002*	*p*=0.003*
	Low-flow suction	3.5 (2.6;4.3)			
	High-flow suction	1.9 (.5;2.5)			
Groups of AGP					
			*p* value between HSP and ADP for no-suction	*p* value between HSP and ADP for low-flow-suction	*p* value between HSP and ADP for high-flow-suction
HSP	No-suction	3.8 (2.1;5.5)	*p*=0.886**	*p*=0.200**	*p*=0.330**
	Low-flow suction	3.0 (2.0;3.8)			
	High-flow suction	1.8 (1.4;2.5)			
ADP	No-suction	3.4 (2.8;5.0)			
	Low-flow suction	4.0 (3.2;4.8)			
	High-flow suction	1.9 (1.6;2.5)			
Groups of cannulas					
			*p* value between no-suction and SAE	*p* value between no-suction and HC11	*p* value between no-suction and HC16
No-suction		3.4 (2.6;5.4)	*p*=1.000**	*p*=0.316**	*p*=0.001**
Low-flow	SAE	3.5 (2.6;4.3)		*p*-value between SAE and HC11	*p*-value between SAE and HC16
High-flow	HC11	2.4 (1.7;3.1)		*p*=0.385**	*p*=0.002**
	HC16	1.9 (1.5;2.5)			*p*-value between HC11 and HC16
					*p*=0.281**

The results (median [25th; 75th percentiles]) for the area of splatter contamination (SCON in %) in a circle 1m around the manikin head according to the three categories of flow rate (nosuction; low-flow-suction with SAE (150l/min); high-flow-suction with HC11 or HC16 (≥250l/min)

Aerosol generating procedures (AGP); air-polishing (ADP); high-speed tooth preparation (HSP: high-speed contra-angle handpiece, turbine); saliva ejector with 6mm diameter (SAE); suction cannula with 11mm diameter (HC11); suction cannula with 16mm diameter (HC16). *Kruskal-Wallis-Test (Bonferroni correction); **Mann-Whitney-U-test

As stated before, a completely elimination of generated aerosols and splatter contamination seems elaborate; our measured contaminated area after no suction for HSP and ADP was without significant difference (*p*=0.886) and in size nearly similar of two DIN A4 paper versus only of one sheet after utilizing HC16 cannulas. Hence, we indicated a continuum of procedure-related aerosol generation for ADP versus HSP for the direction and distance of splatter contamination (Fig. 4). This is in line with the results published recently by Kaufmann et al. [17], which showed contamination of the nearby structures and affirming the value of wearing protective equipment and effective routine infection control in dentistry.

As we failed to show differences between HSP and ADP, we have to hypothesize that the measuring method and definition for spray mist/aerosol will be one of the reasons for it [3, 20]. Kun-Szabo et al. [20] measured the aerosol concentration with spectrometry and identified a more easily controlling of aerosols generated by ultrasonic scaler versus dental turbine. They found that the efficiency of air spray control depends on how exactly the AGP instrument is used during a treatment; when the air spray is frequently directed toward the air of the operatory is the most difficult to control [20]. With a similar measuring method, Kaufmann et al. [17] found that ADP led to greater contamination than ultrasonic. All these results are in line with those from Vernon et al. [3], which was the first study report for aerosolization of active virus as a marker for risk determination in a dental clinic. They found that, compared to a dental turbine, a high-speed contra-angle handpiece reduced settled bioaerosols by 99.72%, 100.00%, and 100.00% for no mitigation, aspiration, and rubber dam, respectively [3]. Yet, the use of rubber dam is neither applicable in ADPs nor during ultrasonic scaling.

In addition to the efforts to improve the effectiveness of intra-/extraoral suction equipment [15, 25, 26], there is also an idea of reducing the amount of fluid spray during AGPs [27]. When HSP and ADP are used according to manufacture/our internal treatment guidelines, we measured lower water/powder fluid for ADP versus HSP—but that is not equivalent to the subjective sensation of the majority of the user and special equipment or techniques to control spray mist have been described [23, 28]. The higher speed and more density of the air spray particle (powder and fluid) of the ADP spray mist leads to a higher contamination of more distant structures. We found this circumstance when low-flow rate suction or no-suction were performed as in the beginning of our treatment simulation for ADP greater size of splatter was measurable (Fig. 4c). Maybe, this could be explained by possible powder rest in the air flow device. However, we found that an intraoral suction with high-flow rate ≥250l/min could slow down these larger particles of ADP, whereat the part of not eliminated splatter physically will drop quicker to the floor or other nearby surfaces. We could measure such effect for ADP according to the significant lower SCON when HC11 or HC16 were utilized (Table 1). This is in line with other findings [19, 28], which recommend HVE at all times and a correct handling of the handpiece angulation to avoid that spray mist will deflect in the direction of the clinician. Still, up to date, the knowledge about the higher contamination risk utilizing ADP devices is limited and higher attention for the control of spray mist and aerosol is recommended when ADP devices were used. A solution will be a suction cannula of HC16 with a funnel-shaped opening (Fig. 1), intraoral positioned nearby the air-polishing handpiece. In a previous study testing this new developed cannula, we found no such anticipated improvement for mitigation spray mist, moreover tended to be too cumbersome to use [8]. Under clinical situation, it is sometimes difficult and time-consuming to handle two bulky instruments (e.g., ADP handpiece and suction cannula) simultaneously. At the same time, sufficient visibility and additional safe support must be maintained when working on two sides. Training to use this two-hand technique safely and optimally is essential. This corresponded with our current observations for all cannula HC16, whereat the smaller straight-line design of the HC11 cannula tends to easily utilizing, especially in the molar region. However, neither a significant difference between HC11 versus HC16 according SCON values were measured (*p*=0.281) nor we detected differences among the three HC16 cannulas as we did not perform any subgroup analysis (inadequate number of tests). Hence, the efficiency of control might depend on how exactly all cannulas are used during a treatment [8]. Unfortunately, in our simulation, we have not always control for optimal position of the cannula—comparable with a clinical situation. Therefore, our study could not provide conclusive results in this respect. Irrespective of the simple and inexpensive methods for the control of spray mist already available [29], sometimes dentists ignore it because of low awareness of health risks, working habits, and economic factors [15, 30].

Different limitations of the current experimental study have to be pointed out. The aim was exclusively to investigate the contamination of splatter with droplets ≥100μm. While these larger droplets will sink to the ground within a few seconds as proofed by the current data, droplets less than 5μm in diameter can remain airborne for hours and can be transmitted by air streams over longer distances [31, 32]. But the method we use is limited as it is not designed to detect droplets smaller than 100μm. Instead, our intention was to measure in a larger area around a manikin head splatter generated during AGPs and the possibility to control it by different dental suction devices. Therefore, we do not claim to investigate the bioaerosol infection risk originating from those small droplets. Nevertheless, airborne droplets of all sizes can carry potentially pathogenic microorganisms like viruses and bacteria and several methods have been described for measuring aerosolization in dentistry, including air particle measurement [26, 33], biological air sampling [34], the culturing of settle plates [35], and detection of fluorescent markers via indirect techniques with coloring the fluid [8, 33]. We know that the use of fluorescent dyes cannot reveal the viability of any biological component, and as we did not measure the fluorescence intensity, only a quantitative analysis of the splatter and deposit distribution was possible. Another limitation of our study was the 2D visualization of the treatment room [8, 36]. Only the horizontal dimension of the area of interest was photographically documented during AGP. The splatter, which settles on vertical surfaces such as furniture of the room, clothes, the face, or face guard, is only conditionally viewable. Also, the manikin head is not a correct anatomical reference with its wide mouth opening and no tongue as an example. Therefore, our in vitro results cannot be transferred 1:1 to a clinical setting. Due to the complexity of airborne disease transmission, it is difficult to quantify the effects of saliva, blood, breathing, coughing, and swallowing patient interaction for calculating the exact risk of aerogene infection of, e.g., SARS-CoV-2 [37]. Especially for this virus, it must be assumed that patients’ saliva and coughing, therefore, will further increase the total amount of splatter contamination with higher risk of aerogene infection [3, 17]. Only splatters and droplets over 100μm in size were with our measurement detected with a high risk that smaller particles were overseen or in such small particles that they will not fall on clinical surfaces [38]. In worst-case scenarios, there can be shown a slightly delayed aerosol particle distribution, e.g., on clinical extremities [3]. At least, the study was performed in a closed room without any air ventilation, so the accumulation of smaller droplets during AGPs could be assumed as higher than in a clinical situation with more movement of the examiner, assistants, or because of open windows or doors which well significant influence the distribution of smaller particles [26, 33]. Although these limitations of our experimental study should be taken into account when trying to draw conclusions for “real” clinical dental treatment, however, the findings help to improve the current understanding of intraoral suction and provide highly reliable and reproducible data.

## Conclusions

Within the limits of the study, according to the current data, it seems impossible to completely eliminate generated spray mist and splatter contamination, and therefore, we strongly recommended to utilizing an HVE with suction cannulas of 16mm diameter for optimized high-flow rate (≥250 l/min) during all AGPs and also to disinfect all surface of patients or operators contact after all AGPs, respectively.

## Declarations

### Disclaimer

DÜRR DENTAL SE did not play a role in the study design, data collection and analysis, decision to publish, or preparation of the manuscript.
